# Creating the evidence base for palliative care in cancer – models and strategies to build research capacity

**DOI:** 10.3332/ecancer.2024.1819

**Published:** 2024-12-12

**Authors:** Eve Namisango, Tonia Onyeka, Nafula Esther, Emmanuel B K Luyirika, Zipporah Ali, Richard A Powell

**Affiliations:** 1 African Palliative Care Association; 2 Pain and Palliative Care Unit, University of Nigeria, Nsukka, Nigeria; 3 IVAN Research Institute, Enugu, Nigeria; 4 Hospice and Palliative Care Association of Nigeria, Keffi, Nigeria; 5 Pain and Palliative Care Unit, Kenyatta National Hospital, Nairobi, Kenya; 6 Formerly of Kenya Hospices and Palliative Care Association, Nairobi, Kenya; 7 Department of Primary Care and Public Health, Imperial College London, London, UK, W12 0BZ

**Keywords:** research, evidence, cancer, research capacity, Africa

## Abstract

**Background:**

The need for palliative care in cancer is highest in resource-limited settings given the high disease burden, which is projected to double by 2050, and late patient presentation. To stimulate service development and ensure care is aligned to patients’ and families’ needs, robust evidence is needed. However, Africa continues to be under-represented globally in evidence development, due to lack of a critical research mass and financial and infrastructure challenges. Despite these limitations, the region is witnessing growth in research for palliative care in cancer. This review aimed to identify models, strategies and practices for building capacity for research and creation of an evidence base for palliative care in cancer in Africa.

**Approach:**

We reviewed grey and published literature to identify models, strategies and practices for building capacity for research and creation of an evidence base for palliative care in cancer in Africa. The findings were summarised using narrative synthesis.

**Findings:**

Models and strategies identified, which are not mutually exclusive, include: community engagement; centres of excellence; knowledge exchange platforms; research networks; practice-based research networks; local collaboration and Global South-to-South partnerships and Global North-to-South partnerships.

**Conclusion:**

The evidence base for palliative care in cancer in Africa is growing in Africa and identifiable models can and are steering this growth.

## Background

Cancer remains a major public health concern and one of the leading causes of death below the age of 70 years globally [1]. In 2020, an estimated 801,392 new cases of cancer and 520,158 cancer-related deaths occurred in sub-Saharan Africa alone [2]. A strong association exists between the incidence and prevalence of cancer and the level of socio-economic development [3]. Developing countries have higher disease burdens and poorer survival rates, mainly attributed to late presentation [4]. A diagnosis of cancer is associated with burdensome symptoms and concerns associated with distress [5].

There is, therefore, a high need for palliative care – defined as ‘a crucial part of integrated, people-centred health services … relieving serious health-related suffering, be it physical, psychological, social, or spiritual’ – for people living with cancer and their families to address their symptoms and concerns and improve their quality of life [6]. The majority of adults in need of palliative care (76%) live in low-and-middle-income countries (LMICs) [7]. Among children and young people, the need for palliative care also remains high, with HIV and cancer making the largest contribution [8]. Tailoring palliative care to the preferences and cultural backgrounds of cancer patients and their families fosters a sense of involvement and empowerment, increasing trust and overall satisfaction with the healthcare experience. By following evidence-based guidelines, palliative care professionals can optimise patient outcomes, improve symptom management and enhance the overall quality of life for patients with serious illnesses [9].

Whilst there have been advances in palliative care service development in Africa [10], existing provision is incommensurate with need. The pillars for palliative care service development are education, policy, service delivery, medicine availability [11] and research [9]. To deliver care that focuses on patients and their families holistically, rigorous research is critical to underpinning and driving forward service development, evaluation, quality improvements and care delivery.

This evidence is essential for multiple reasons. First, for delivering high-quality, consistent and patient-centred care, helping healthcare providers make informed decisions about treatment options for patients that recognises their unique needs, preferences and values, ensuring the care provided is individualised. Second, it provides a foundation for ethical decision-making, helping healthcare providers navigate challenging ethical dilemmas by relying on research to inform decisions that align with the principles of beneficence and non-maleficence [9]. Third, it ensures the prioritization of proven interventions, ensuring providers use resources efficiently and patients receive appropriate care without unnecessary or ineffective interventions. Fourth, by regularly reviewing and updating practices based on new evidence, it supports a culture of continuous quality improvement. This ensures palliative care practices remain up-to-date, effective and aligned with the evolving understanding of best practices in the field.

The global expansion of the palliative care evidence base has been notable but there persists an underrepresentation from LMICs [12, 13], including in the field of cancer. In Africa, a number of research studies have focused on improving the management of symptoms associated with cancer and its treatments (i.e., pain, fatigue, nausea, sleep disturbance and so on), digital technology [14] and assessment tools [15]. However, many gaps persist. Historically, palliative care research in Africa has suffered from short-term, project-focused initiatives, insufficient funding, reliance on a few individuals, communication challenges and an imbalance in North-South partnerships, often dominated by Northern partners [16]. Also, national research endeavours across the continent are sparse [17]. National palliative care research agendas have been established [18, 19] but the extent of their implementation and impact has not been evaluated. Moreover, disparities exist in the areas of palliative care research; studies on psychosocial and spiritual issues and end-of-life care and studies testing nursing interventions to improve patient outcomes are grossly lacking [20].

Underpinning these limitations and challenges is the need to establish a lasting foundation for building research capacity, including skills and knowledge, in the region [21]. To foster the ongoing development of the evidence base for palliative care in cancer, we aimed to identify models and strategies for building research capacity and creating an evidence base for palliative care in cancer in Africa.

## Methods

We reviewed grey and published literature on models, practices and strategies to build research capacity in Africa. We synthesised the literature narratively.

## Findings

The models and strategies found, which are not mutually exclusive, include: community engagement; centres of excellence; knowledge exchange platforms; research networks; practice-based research networks (PBRNs); local collaboration and Global South-to-South partnerships and Global North-to-South partnerships.

### Community engagement

The current public health approach to palliative care promotes the concept of ‘compassionate communities’ and demands that palliative care researchers engage with communities, not as experts or custodians of knowledge, but as co-creators, in partnership with communities when engaged in research [22].

Research initiatives that involve local communities are more likely to be sustainable in the long term. By building capacity within the community and fostering local ownership of research projects, interventions or programs are more likely to be integrated into existing community structures and continue beyond the duration of the research study. Overall, involving local communities in the research process is not only ethically imperative but also enhances the quality, relevance and impact of research initiatives by ensuring they are culturally sensitive, responsive and meaningful to the communities they seek to serve.

### Centres of excellence

Centers of excellence in oncology play a vital role in advancing the evidence base for palliative care in cancer in several ways. First, they often have dedicated research teams focusing on various aspects of oncology, including palliative care. By allocating financial and human resources to palliative care research, these centers can generate valuable data and insights into effective interventions, patient preferences and outcomes.

Centers of excellence typically involve collaboration among various specialists, including oncologists, palliative care physicians, nurses, psychologists and social workers. This interdisciplinary approach fosters the integration of palliative care into the overall cancer care continuum and encourages research that considers the holistic needs of patients and their families. These centers also give an opportunity for the conduct of clinical trials to evaluate new treatments, interventions and care models. By including palliative care components in these trials, researchers can gather empirical evidence on the efficacy and impact of different palliative care approaches, such as symptom management, psychosocial support and end-of-life care strategies. Centers of excellence often serve as hubs for education and training for healthcare professionals, researchers and students. By incorporating palliative care education into their programs, they ensure the next generation of clinicians and researchers are equipped with the knowledge and skills necessary to advance the field and contribute to the evidence base through their own research efforts.

At present, Nigeria has six designated oncology centres of excellence at teaching hospitals which are tertiary health institutions, each located in one of the six geopolitical regions of the country. This is in addition to other government-owned and private cancer centres, such as the NSIA-LUTH Cancer Centre, a five-star cancer treatment facility that is a joint venture between the Nigeria Sovereign Investment Authority and the Lagos State University Teaching Hospital. The Uganda Cancer Institute, on the other hand, runs fellowship programmes for clinicians and, as part of their training, they must undertake a research project. The facility-based palliative care teams encourage fellows to consider palliative care-centred research questions as part of the in-house strategy to use research to improve patient care.

In countries experiencing political instability like Sudan, groups of health professionals attend Fellowships in Palliative Medicine, for example, the Indian Palliative Medicine program, which includes a research component. Other members of the Palliative Care Team members have completed the paediatric Echo program and new members continue to enroll. Again, they are introduced to pediatric palliative care research and encouraged to undertake research.

### Knowledge exchange platforms

Conferences are one of the major platforms inspiring practitioners to engage in research and disseminate and share their findings. The African Organization for Research and Training in Cancer (AORTIC) is one of the major conferences at which knowledge generators, users and brokers commonly meet to deliberate on research agendas, share best practices and to nurture partnerships and networks. The African Palliative Care Association (APCA) commonly runs cancer and palliative care co-hosted conferences to strengthen the evidence base of palliative care in cancer [23, 24]. Palliative care leverages on cancer knowledge exchange platforms that have high-profile visibility, with examples including the International Hepato-Pancreatico-Biliary Association and Hepato-Pancreato-Biliary Association of Southern Africa global conference held in Cape Town in 2024^1^. Palliative care was allotted a day at this high-profile meeting and key players showcased cutting-edge findings from implementation and operational research in Africa.

### Research networks

Research networks in palliative care are collaborative platforms where researchers, healthcare professionals and organizations work together to pool and share knowledge, resources, data and expertise, thereby enabling larger, more comprehensive studies than individual entities could undertake alone. One such network is the African Palliative Care Research Network (APCRN), formed under the APCA to bring together key stakeholders in the region to engage in activities geared towards building the evidence base for palliative care [25]. The APCRN has 300 members and university-affiliated hub coordinators in the four regions of the continent: West, East, North and Southern Africa. The APCRN has achieved notable progress by publishing five manuscripts and organising five capacity-building workshops for emerging researchers. These are held during the APCA tri-annual conferences [26] and facilitate knowledge sharing and career advice among current researchers. Furthermore, APCRN’s educational webinars provide a platform for professionals to discuss projects, share findings and address methodological questions, promoting shared learning. This model holds promise for capacity-building and knowledge translation in African palliative care research.

The Africa Survivorship Working Group (ASWG), a multi-stakeholder special interest group and research network affiliated with the Africa Cancer Research and Control ECHO of the National Cancer Institute [27], emerged out of the desire to analyse the current situation of cancer survivorship on the African continent [27, 28]. A network of researchers, clinician experts, policymakers and cancer survivors, it seeks to identify gaps in knowledge and priorities for research. The local expertise within the ASWG ensures that studies are designed and implemented with sensitivity to cultural norms, beliefs and practices, thereby enhancing the relevance and impact of research findings. Research networks, through the generation of robust evidence on the effectiveness, cost-effectiveness and benefits of palliative care interventions, can influence policymakers and stakeholders to prioritise palliative care as an essential component of healthcare systems in Africa. For example, the evidence provided by ASWG through rigorous research influenced the inclusion of survivorship care in the revised National Cancer Control Plan for Nigeria.

### Practice-based research networks

PBRNs are collaborative groups of healthcare professionals and practices that work together to conduct research within real-world healthcare settings [29]. These networks are characterised by their focus on generating evidence for use in everyday care by incorporating research into routine clinical practice and involve clinicians actively participating in the research process.

The ICON-3 Practice Based Research Network of the IVAN Research Institute, affiliated with the Nigeria Implementation Science Alliance, is the first large PBRN in Nigeria. It is currently comprised of 12 secondary comprehensive health facilities, 12 community centers and 12 tertiary academic centers across the six geopolitical regions of Nigeria. It is focused on research in various clinical disciplines and has a national network of investigators engaged in rigorous research and clinical trials in palliative care, cardiology, neurology, oncology, hematology, obstetrics and gynecology. In building an African research evidence base in palliative care, these networks facilitate cross-institutional collaboration, enabling the pooling of data, standardization of research methodologies and fostering a collective effort to address the unique challenges and healthcare needs in the region, ultimately enhancing the quality and relevance of palliative care research in Africa.

### Local collaboration and global South-to-South partnerships

Meaningful partnerships is one of the key strategies that can help foster research in palliative care in cancer. These partnerships are underpinned by active engagement and involvement in prioritising research questions, capacity building and joint ownership for the study outputs [21]. Collaborations between African healthcare institutions, non-governmental organizations (NGOs) and research organizations can play a crucial role in executing effective palliative care research strategies in several ways. Such ways include providing opportunities for networking, sharing resources and capacity-building opportunities. The AORTIC is a trilingual (English, Portuguese and French) non-profit organization committed to advancing cancer control and palliative care in Africa. Among its goals for cancer control in the region is the facilitation of research initiatives.

### Global North-to-South partnerships

These kinds of partnerships play a crucial role in advancing palliative care research in Africa by fostering collaboration, building capacity, promoting cultural sensitivity and generating evidence to inform policy and practice. These partnerships could be between hospitals, organizations, educational institutions, national ministries of health, nations and individuals.

One of the objectives that the Cancer Moonshot Program of the First Couple of the United States of America, President Joe and Dr Jill Biden, sets out to achieve is to assist in developing and implementing new, practical technological interventions, fostering clinical trial development, establishing research centers and enhancing institutional capacity for global cancer research in African countries [30]. This is made possible through funding of biomedical research of African researchers through several grant mechanisms by institutions like the National Cancer Institute and the National Institutes of Health. Initiatives like the Global Alliance program of St. Jude’s Childrens Research Hospital (a Center of Excellence for Influenza Research and a World Health Organization Collaborating Center for Childhood Cancer) have created a network of interactive institutions focused on reducing the cancer gap worldwide. This alliance facilitates the exchange of knowledge, skills and best practices in palliative care research. Researchers from Africa can benefit from training, mentorship and capacity-building opportunities provided by their counterparts in the Global North, enhancing their research capabilities and expertise. Collaborative research partnerships can provide access to resources, funding and infrastructure that may be lacking in Africa. This includes access to research grants, equipment, technology and facilities for conducting high-quality research in palliative care. An example is the partnership between the American Cancer Society (ACS) and Nigeria, Uganda, Kenya and Ethiopia, where the success of the Pain-Free Hospital Initiative through training of clinicians on opioids and the provision of access to affordable moderate opioids (like morphine) has resulted in drastically reduced pain scores in cancer patients, improved clinical outcomes and contributed to the body of pain research in Africa. Similarly, the African Cancer Coalition, a growing body of over 100 oncology experts representing 13 countries in Sub-Saharan Africa, is partnering with the ACS, the National Comprehensive Cancer Network and the Clinton Health Access Initiative to develop standard cancer treatment guidelines for use in Sub-Saharan Africa. This collaboration has the potential to catalyse research advancement, strengthen healthcare systems and improve cancer outcomes in the region through coordinated efforts to standardise care, build capacity and generate evidence for informed decision-making.

The American Society of Clinical Oncology (ASCO) offers a variety of research grants to palliative care researchers from LMICs through the International Innovation Grant to address global disparities in cancer research and care. By providing these funding opportunities, ASCO supports the development of research capacity, fosters innovation and improves cancer outcomes in regions where resources and infrastructure may be limited. Global Partners in Care (GPIC) is a US-based NGO that prioritises advancing research and expanding learning opportunities to improve access to quality palliative care globally. GPIC collaborates with universities and research institutions to generate evidence for assessing needs, implementing policies and monitoring progress in global palliative care. Working in conjunction with the APCA, national associations and African universities, GPIC offers internships for American graduates to volunteer time and expertise in assisting individuals in African institutions with projects and research endeavors. Bio Ventures for Global Health spear-headed the creation of the African Access Initiative, a public-private partnership utilising data produced by African hospitals and governments at the national level to empower African investigators to lead innovative, diverse and inclusive clinical trials.

### Key transferable learning points

- Building an evidence base for African palliative care and cancer is imperative to inform policy change, drive service development, quality improvement and, ultimately, patient care and wellbeing.
- Building the human capacity (including skills and knowledge) to generate evidence on the continent is critical to its longer-term, sustainability.

## Conclusion

The need for a robust evidence base to underpin palliative cancer treatment is as urgent as ever. A number of models and strategies for fostering this evidence base exist, ranging from effective community engagement to Global North-Global South partnerships. Underpinning all has to be a spirit of collaboration, and mutual and shared learning.

## Conflicts of interest

All authors have no conflict of interest to declare.

## Funding

This paper did not receive any funding.

## Disclaimer

RAP is supported by the National Institute for Health and Care Research (NIHR) Applied Research Collaboration Northwest London. The views expressed are those of RAP and not necessarily those of the NIHR or the Department of Health and Social Care.

## Footnotes

1
https://www.ihpba2024.org/
